# A key ‘foxy’ aroma gene is regulated by homology-induced promoter indels in the iconic juice grape ‘Concord’

**DOI:** 10.1038/s41438-020-0304-6

**Published:** 2020-04-18

**Authors:** Yingzhen Yang, José Cuenca, Nian Wang, Zhenchang Liang, Honghe Sun, Benjamin Gutierrez, Xiaojun Xi, Jie Arro, Yi Wang, Peige Fan, Jason Londo, Peter Cousins, Shaohua Li, Zhangjun Fei, Gan-Yuan Zhong

**Affiliations:** 1 US Department of Agriculture-Agricultural Research Service, Grape Genetics Research Unit, Geneva, NY USA; 2 Beijing Key Laboratory of Grape Sciences and Enology, Laboratory of Plant Resources, Institute of Botany, Chinese Academy of Sciences, Beijing, China; 3 Boyce Thompson Institute for Plant Research, Cornell University, Ithaca, NY USA; 4 US Department of Agriculture-Agricultural Research Service, Plant Genetic Resources Unit, Geneva, NY USA; 5 Forestry and Pomology Research Institute, Shanghai Academy of Agricultural Sciences, Shanghai, China; 6 University of Chinese Academy of Sciences, Beijing, China; 7 E. & J. Gallo Winery, Modesto, CA USA; 8 US Department of Agriculture–Agricultural Research Service, Robert W. Holley Center for Agriculture and Health, Ithaca, NY USA; 9 Present Address: Centro de Citricultura y Producción Vegetal. Instituto Valenciano de Investigaciones Agrarias, Moncada, Valencia, Spain; 10 Present Address: College of Horticulture and Forestry, Huazhong Agricultural University, Wuhan, Hubei China

**Keywords:** Genome, Gene regulation

## Abstract

‘Concord’, the most well-known juice grape with a parentage of the North American grape species *Vitis labrusca* L., possesses a special ‘foxy’ aroma predominantly resulted from the accumulation of methyl anthranilate (MA) in berries. This aroma, however, is often perceived as an undesirable attribute by wine consumers and rarely noticeable in the common table and wine grape species *V. vinifera*. Here we discovered homology-induced promoter indels as a major genetic mechanism for species-specific regulation of a key ‘foxy’ aroma gene, anthraniloyl-CoA:methanol acyltransferase (*AMAT*), that is responsible for MA biosynthesis. We found the absence of a 426-bp and/or a 42-bp sequence in *AMAT* promoters highly associated with high levels of *AMAT* expression and MA accumulation in ‘Concord’ and other *V. labrusca*-derived grapes. These promoter variants, all with direct and inverted repeats, were further confirmed in more than 1,300 *Vitis* germplasm. Moreover, functional impact of these indels was validated in transgenic *Arabidopsis*. Superimposed on the promoter regulation, large structural changes including exonic insertion of a retrotransposon were present at the *AMAT* locus in some *V. vinifera* grapes. Elucidation of the *AMAT* genetic regulation advances our understanding of the ‘foxy’ aroma trait and makes it genetically trackable and amenable in grapevine breeding.

## Introduction

Grapevine (*Vitis spp*.) is one of the most important fruit crops in the world. Among ~60 *Vitis* species, *Vitis vinifera* L. is widely cultivated as wine, table, and raisin grapes. *Vitis labrusca* is a wild North American grapevine species^1^ with a long history of being used for interspecific hybridization with *V. vinifera* for the development of juice, table, and wine grapes^2^. ‘Concord’, developed in 1849, is the most well-known juice grape cultivar^3^. Frequently considered as a *V. labrusca* cultivar, ‘Concord’ has about 1/3 of *V. vinifera* in its pedigree^4,5^. The success of ‘Concord’ as the leading juice grape is largely attributed to its productivity, disease resistance, as well as unique ‘foxy’ flavors and nutritional properties^6,7^. ‘Foxy’ aroma is a complex term to describe the unique, earthy, and sweet muskiness present in most *V. labrusca* and derived hybrid grapes. It is this ‘foxiness’ that makes ‘Concord’ grape very popular for the production of non-fermented juice and jellies^8^. However, ‘foxy’ aroma is often perceived to be an undesirable attribute for wine grapes and rarely noticeable in *V. vinifera* and most other *Vitis* species^9,10^. In interspecific wine grape breeding involving introgression of adaptive *V. labrusca* germplasm into *V. vinifera*, ‘foxy’ aroma is treated as an undesirable trait to be eliminated through tasting and/or biochemical evaluation.

The characteristic ‘foxy’ aroma of ‘Concord’ grapes is likely a result of several key volatile compounds with methyl anthranilate (MA) being the most important^9–15^. The biosynthesis of MA in grapes involves the anthraniloyl-coenzyme A (CoA):methanol acyltransferase (AMAT) that catalyzes the formation of MA from anthraniloyl-CoA and methanol, resulting in an ester of anthranilic acid^13^. In maize and strawberry, an anthranilic acid methyl transferase (AAMT) can synthesize MA directly from anthranilic acid^16,17^. However, the involvement of AAMT in MA synthesis has not been biochemically validated in *Vitis* species.

Two *V. vinifera* reference genomes, based on a nearly homozygous line PN40024 derived from ‘Pinot noir’ and a highly heterozygous ‘Pinot noir’ clone ENTAV 115, respectively, were released in 2007^18,19^. Additional *Vitis* genome projects, mostly involving *V. vinifera*, have been pursued in the last decade^20–33^. However, no genomes of *V. labrusca* cultivars have been reported. To provide genomic resources for elucidating the genetic control of ‘foxy’ aroma and other fruit traits characteristic to ‘Concord’ and other grapes with *V. labrusca* genetic background, we produced a draft genome and berry transcriptomes of ‘Concord’. We compared the genomic and transcriptomic profiles of ‘Concord’ with that of *V. vinifera* and discovered two large promoter sequence variants that were likely responsible for the differential expression of the *AMAT* gene between *V. labrusca* and *V. vinifera*. We further confirmed this causal relationship by analyzing the genomic and transcriptomic profiles of the *AMAT* gene and the accumulation of MA compound in 50 *Vitis* germplasm accessions. The diagnostic promoter variants were further validated in more than 1300 *Vitis* germplasm accessions. Furthermore, the functional impact of the promoter sequence variants on gene expression was validated in transgenic *Arabidopsis*. Additional structural variation in the *AMAT* promoter and coding regions were observed in *V. vinifera* and its progenitor species *V. sylvestris*, suggesting the presence of additional layers of regulation of the *AMAT* gene in common wine and table grapes. Interestingly, all these promoter sequence variants had direct and inverted repeats at the indel boundaries, suggesting that they were likely generated by a homology-based mechanism such as replication slippage. This work significantly advances our understanding of how *AMAT* is regulated as a key ‘foxy’ aroma gene in ‘Concord’ and other grapes and provides genomic resources and knowledge for tracking and manipulating this important aroma trait in a grape breeding program.

## Results

### ‘Concord’ genome sequence and pedigree

We used a whole-genome shotgun strategy for ‘Concord’ genome sequencing and assembly (Supplementary note 1 and Tables S1–S8). Based on the frequency distribution of 21-mers (Supplementary note 7, Fig. S1), the ‘Concord’ genome was estimated to be 499 Mb, similar to the *V. vinifera* reference genomes^18,19^. De novo assembly of the Illumina reads, however, resulted in a larger ‘Concord’ genome assembly of 570.8 Mb, likely due to its high heterozygosity (Supplementary note 7, Fig. S1). A total of 25,499 protein-coding genes were predicted in the ‘Concord’ genome and 12,659 gene pairs were defined between ‘Concord’ and the PN40024 reference genome (Table S6). About 39% of the gene pairs showed substantial low collinearity in their 3-kb promoter regions (Tables S6 and S9), suggesting the presence of large structural variations in these promoter pairs.

‘Concord’ was presumably produced by a cross between ‘Catawba’ and an unknown female parent, likely a wild *V. labrusca*. ‘Catawba’ is, in turn, a hybrid between ‘Semillon’, a *V. vinifera*, and another unknown wild American grape^5^. These pedigrees were largely supported by our analysis of the genotypic concordance of 13 million SNPs among ‘Concord’, ‘Semillon’, ‘Catawba’, and four *V. labrusca* accessions (Supplementary note 1 and Table S10).

### Berry transcriptome comparison between ‘Concord’ and *V. vinifera*

**Fig. 1:**
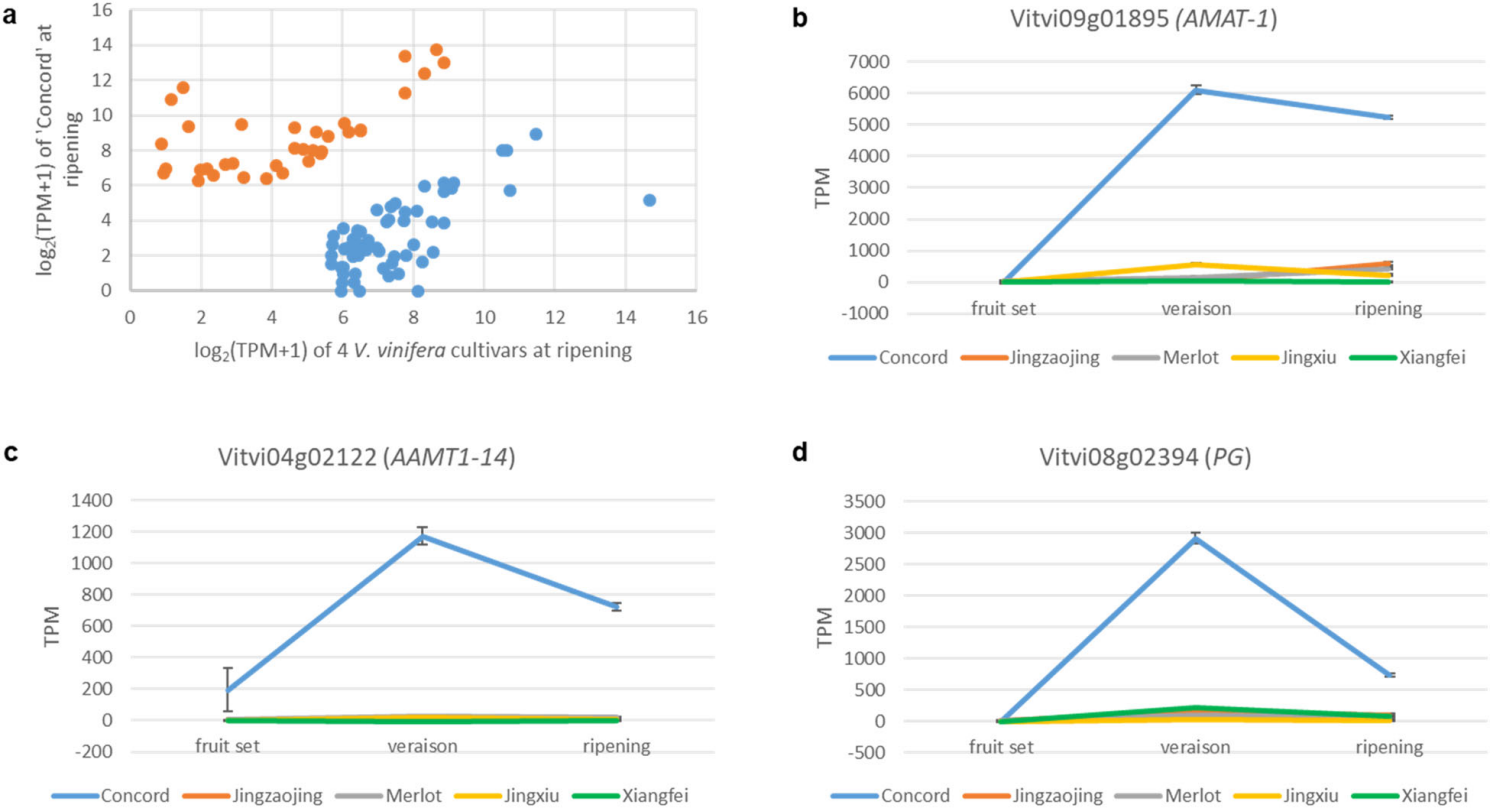
Differentially expressed genes (DEGs) between ‘Concord’ and *V. vinifera* at veraison and ripening stages. **a** DEGs with more than 5-fold higher (orange, *n* = 35) or lower (blue, *n* = 58) expression in ‘Concord’ than in four *V. vinifera* cultivars at both veraison and ripening stages (TPM >= 50). Expression profiles of *AMAT-1* (*Vitvi09g01895*) (**b**), *AAMT1-14* (*Vitvi04g02122*) (**c**), and one polygalacturonase (*PG*) gene (*Vitvi08g02394*) (**d**) at three berry developmental stages in ‘Concord’ and four *V. vinifera* cultivars

Compared with *V. vinifera* grapes, ‘Concord’ has many unique fruit traits for which genetic control is yet to be revealed. To identify genes with differential expression patterns during berry development between ‘Concord’ and *V. vinifera*, RNA-Seq data from fruit set, veraison, and ripening of ‘Concord’ and four *V. vinifera* cultivars, ‘Merlot’, ‘Xiangfei’, ‘Jingzaojing’ and ‘Jingxiu’, were examined (Table S11). We focused on genes with differential expression between different fruit stages as well as between ‘Concord’ and *V. vinifera* cultivars. A total of 996 genes showed expression levels of at least 50 TPM (Transcripts per million reads) at the fruit set or veraison stage in either ‘Concord’ or *V. vinifera* and at least 5-fold difference between fruit set and veraison stages (Table S12). Ninety three were identified as DEGs at both veraison and ripening stages between ‘Concord’ and *V. vinifera* cultivars (fold change ≥ 5 and adjusted *p* ≤ 0.05) with 35 being expressed higher in ‘Concord’ (Fig. 1a and Table S12). Among these 35 DEGs, *AMAT* and 10 others showed low collinearity in their 3-kb promoter regions between ‘Concord’ and *V. vinifera* (Table S6).

MA is one of the major compounds for the characteristic ‘foxy’ aroma in ‘Concord’ and other cultivars with the *V. labrusca* pedigree. Three genes have been demonstrated to encode enzymes which can synthesize MA from anthranilic acid, including *AMAT* from ‘Concord’^13^, *AAMT1* from maize^17^ and *FanAAMT* from strawberry^16^. A few *AMAT*-like genes exhibited significant higher expression in ‘Concord’ than in *V. vinifera* at both veraison and ripening stages (Table S13). The *AMAT* gene^13^ (Vitvi09g01895) had expression levels in ‘Concord’ of more than 5000 TPM, 32 and 16 folds of that in *V. vinifera* at veraison and ripening, respectively (Fig. 1b). One *AAMT1*-like gene, Vitvi04g02122, had expression over 1000 TPM at veraison in ‘Concord’ but less than 20 TPM in *V. vinifera* (Fig. 1c). The extremely high expression levels of *AMAT* in ‘Concord’ berries suggested that it is likely the major contributor to the high MA level in ‘Concord’.

One other prominent berry trait of ‘Concord’ and other *V. labrusca* grapes is ‘slip-skin’ which refers to that skin can be slipped off easily from the flesh. Pectin is the main polymer that binds cell walls of flesh tissue right below the hypodermal layer of berry skin. Pectin is modified and disassembled during fruit softening and ripening^34^. Polygalacturonase (PG) is a pectin depolymerase and its activity accompanies many plant development processes, particularly those that require cell separation^35^. The expression levels of one *PG* gene, Vitvi08g02394, went up more than 10 times from fruit set to veraison/ripening stages in both ‘Concord’ and *V. vinifera* indicating its role in berry ripening (Fig. 1d). However, it was expressed 24- and 11-fold higher at the veraison and ripening stages, respectively, in ‘Concord’ than in *V. vinifera*. The high expression levels of this *PG* gene at both veraison and ripening stages in ‘Concord’ could be the main cause of its ‘slip-skin’ phenotype.

Among other DEGs, ‘Concord’ had much higher expression for three *wax2*-like genes which might explain its higher content of berry skin wax^36–38^, one gene (Vitvi10g00027) involved in thiamine (vitamin B1) synthesis, and two glutathione S-transferase genes (Tables S12 and S13). On the other hand, some genes were expressed much higher in *V. vinifera* than in ‘Concord’, including certain *MLO* (Mildew Locus O) and glutelin type-A genes (Tables S12 and S13). The significance of these DEGs for manifestation of relevant traits remains to be investigated.

### Large sequence variations discovered at the *AMAT* locus

**Fig. 2:**
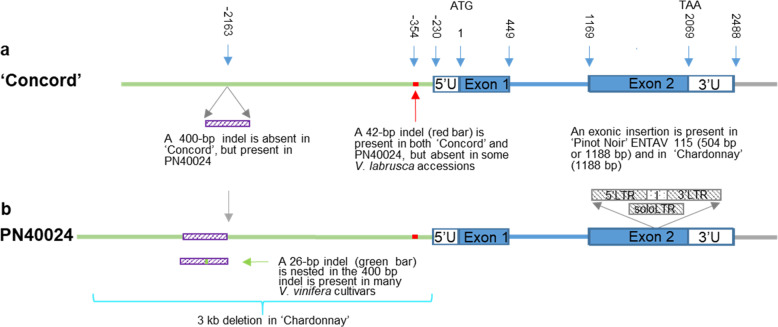
Schematic representation of the *AMAT* locus with major indels. **a** *AMAT* locus in ‘Concord’. The promoter and transcribed regions were respectively colored in green and blue. Based on the RNA-Seq data and cDNA cloning of berry samples, two exons (blue boxes) and one intron (blue line) were defined and their relative positions were marked, with “A” in the ATG start codon as position 1. The 5′ and 3′ untranslated regions were labeled as “5′U” and “3′U”, respectively. A 400-bp (or 426-bp) fragment was absent in ‘Concord’ (purple box) but was present in PN40024 (or other *V. vinifera* cultivars). A 42-bp fragment from −354 to −313 (red bar) in the promoter region was absent in some *V. labrusca* accessions. **b** *AMAT* locus in the PN40024 reference genome. The structure is similar as in ‘Concord’ except the presence of 400-bp sequence. A 3-kb fragment in the promoter region, indicated with an aqua-colored bracket, was deleted in one *AMAT* allele in ‘Chardonnay’ clone I10V1 and some other *V. vinifera* accessions^30^^,31,33^. Two types of insertions (gray boxes) were found in exon 2 at the exact same position, 504 bp (a soloLTR) in ‘Pinot Noir’ clone ENTAV 115^19^ and 1188 bp (a TRIM with 5′LTR, internal sequence and 3′LTR) in the second *AMAT* allele of ‘Chardonnay’ I10V1 and some other accession^29–32^

While MA is a key contributor to the ‘foxy’ aroma in grapes of *V. labrusca* origin and can be produced by AMAT from anthraniloyl-CoA and methanol^13^, little is known about the molecular mechanism underlying the regulation of *AMAT* expression, and thus MA accumulation. RNA-Seq data and sequencing of ‘Concord’ *AMAT* RT-PCR products both revealed that the gene contained two exons and one intron, similar to the published data^13,23^ (Fig. 2 and Table S14). However, presence or absence variation of a large promoter sequence of *AMAT* was uncovered between ‘Concord’ and PN40024 (Fig. 2 and Table S15). This variant contributed to the low collinearity observed in the 3-kb gene-pair promoter analysis of *AMAT* (Table S6). Compared with PN40024, ‘Concord’ lacked a 400-bp sequence about 2.1 kb upstream of the ATG start codon (Fig. 2). We further compared the ‘Concord’ *AMAT* locus with several published contigs of *V. vinifera* cultivars aligned to the *AMAT* region^19,28,30–33^. Compared to ‘Concord’, all examined *V. vinifera* contigs had a 426-bp sequence in their *AMAT* promoters, which was in contrast with the obersered 400 bp in the reference genome PN40024, suggesting that a 26-bp sequence was absent in PN40024 (Fig. 2 and Table S15). Some of these *V. vinifera* contigs also showed deletion of a 3-kb sequence in their *AMAT* promoter regions. Further, large insertions of 504 bp or 1188 bp were found in the 2nd exon of the *AMAT* coding region in ‘Pinot noir’ ENTAV 115 and some other contigs (Fig. 2 and Supplementary note 7, Fig. S2).

We examined the status of the promoter indels of 3 kb, 426 bp, and 26 bp and the exon 2 insertion in 50 *Vitis* germplasm accessions, including many with *V. labrusca* in their pedigrees and several wild species (Table S16 and Supplementary note 2). For the 10 *V. labrusca* accessions, nine were heterozygous for the 426-bp indel (noted as 0/426 in Table S16) and one was homozygous for the presence of the sequence (426/426). For the 33 *Vitis* hybrids, most were *V. labrusca*-derived. They formed four groups based on their 426-bp and 26-bp indel genotypes. The first group had eight hybrids, which lacked the 426-bp sequence (0/0, 0/“-”, or “-”/“-” where “-” represents no detection of the *AMAT* copy in that region). This group included well-known juice grapes ‘Concord’, ‘Catawba’ and ‘Niagara’. The second group had six hybrids with the genotype of 0/400. The third group had eight hybrids with the genotypes of 0/426, while the fourth group had 11 hybrids with the genotype of 426/426 or 426/“-”. All four wild *Vitis* species had the genotype of 426/426. Three *V. vinifera* cultivars, ‘Pinot noir’, ‘Merlot’ and ‘Semillon’, had the genotype of 426/426, 426/“-” and “-”/“-”, respectively. It was a surprise that no *AMAT* locus was detected in ‘Semillon’. For ‘Canada Muscat’, ‘Niagara’ and ‘Merlot’, they lacked one *AMAT* copy in this region due to the 3-kb promoter deletion (Table S16). But for ‘Semillon’, the deletion is likely due to the loss of an undefined size encompassing the *AMAT* locus beyond the 3-kb promoter region, as supported by the fact that the *AMAT* coding region could not be amplified either. The exon 2 insertion was found in ‘Pinot noir’ and 5 *Vitis* hybrids (Table S16).

RT-PCR products of *AMAT* ORFs for ‘Concord’, ‘Catawba’, ‘Caco’, ‘Alba’, ‘Barry’ and *V. vinifera* ‘Merlot’ representing different groups of the 50 germplasm accessions were analyzed. The ORF sequences of ‘Concord’ and ‘Catawba’ were the same, except that at position 414 ‘Concord’ had both A and G while ‘Catawba’ had only G (Table S14), suggesting that ‘Concord’ is heterozygous for the *AMAT* locus with one of the *AMAT* alleles from ‘Catawba’. No major structural variation was found in the ORFs sequenced for these cultivars except ‘Caco’ which had one allele with a 4 bp frameshift insertion.

### Promoter indels and their impacts on *AMAT* expression

*AMAT* expression in the ripening berries of ‘Concord’, ‘Catawba’, ‘Semillon’, ‘Merlot’ and 37 other *Vitis* accessions was measured by quantitative RT-PCR (qRT-PCR) (Table 1 and Table S16). Relative expression levels in a dozen cultivars were also analyzed by RNA-Seq data at the veraison stage (Tables S13 and S16). ‘Merlot’ was used as the baseline for expression comparisons among different germplasm accessions. ‘Concord’ showed about 2-fold expression of ‘Catawba’ while ‘Semillon’ had no *AMAT* expression. Absence of the 426-bp sequence appeared to be associated with high *AMAT* expression (Table 1 and Table S16). Hybrids with absence of one or both copies of the 426- or 400-bp sequence (noted as 0/0, 0/426 or 0/400) on average had 6 to 8-fold higher *AMAT* expression than those with the sequence (e.g. 426/426). In agreement, *V. vinifera* and other non-*V. labrusca* species all carried the 426- or 400-bp sequences in the *AMAT* promoters and showed low levels of *AMAT* expression (Table 1 and Table S16). However, there were some exceptions. *V. labrusca* ‘Alba’ and a few hybrids had the 426 bp but exhibited similar or higher *AMAT* expression compared to ‘Concord’ or ‘Catawba’ (Table S16). Interestingly, absence of a 42-bp sequence in the TATA box region was found in both *AMAT* alleles (0/0) of ‘Alba’ and one other *V. labrusca* accession (Fig. 2 and Table S16). This 42-bp sequence was in heterozygous status in those hybrid accessions (0/42 or 0/“-”) showing relatively high levels of *AMAT* expression (Table S16).

**Table 1:** *AMAT* expression, MA accumulation and status of various *AMAT* indels in ‘Concord’, ‘Catawba’, ‘Semillon’, ‘Merlot’, and other germplasm accessions

Cultivar or germplasm	Pedigree (from NPGS, GRIN-Global, USA)	Indel status^a^		Relative *AMAT* expression		Relative MA content		3 kb promoter deletion (Yes:Y; No:N)	TRIM Insertion in exon 2 (Yes:Y; No:N)
		426 bp indel (position ‘−2595’–> ‘−2164’)	42 bp indel (position ‘−354’–> ‘−313’)	Mean^b^	SE	Mean^b^	SE		
'Concord'	Vitis hybr., *V. labrusca* x Catabaw	0/0	42/42	28.08	0.88	58.38	5.25	N	N
'Catawba'	Vitis hybr., *V. labrusca* x *V. vinífera*	0/“-“	42/“-“	16.37	2.72	1.51	0.10	N	N
'Semillon'	*V. vinifera*	”-“/“-“	”-“ / “-“	0.01	0.00	N.A	N.A	N	N
'Merlot'	*V. vinifera*	426/“-“	42/“-“	1.00	0.20	1.00	0.38	Y	N
Hybrid group 1 (7 hybrids)	hybrids involving *V. labrusca*	0/0 or 0/“-“	42/42	19.56	3.18	18.42	7.55	5 N, 2Y	7 N
Hybrid groups 2 and 3 (14 hybrids)	hybrids involving *V. labrusca*	0/400 or 0/426	42/42 or 0/0	12.80	2.58	3.66	1.75	14 N	13 N, 1Y
Hybrid group 4 (11 hybrids)	hybrids involving *V. labrusca*	426/426 or 426/“-“	42/42, 0/42, 0/“-“ or 42/“-“	2.53	1.37	1.148	0.50	9 N, 2Y	7 N, 4Y
*V. vinifera* and others (5 species, 7 accessions)	Non-*V. labrusca* species	426/426 or 426/“-“	42/42, 42/“-“ or “-“/“-“	0.71	0.41	1.23	0.57	6 N, 1Y	6 N, 1Y
*V. Labrusca* (9 accessions)^c^	*V. labrusca*	0/426	42/42, 0/42, 0/0	N.A	N.A	36.90	10.24	N	N

^a^Indel positions are illustrated as in Fig. 4; two alleles are separated by “/“ and each allele status is indicated by presence of a sequence (426 bp, 400 bp, or 42 bp) or absence (0); “-“ means an allele which was not detected at that region

^b^*AMAT* expression and MA content were calculated as folds of change in relative to that of ‘Merlot’

^c^‘Alba’ was not included in this list due to lack of its MA content assay resulting from poor fruit set; some of the accessions might not be true V. labrusca (Supplementary note 2)

We further surveyed more than 1300 accessions belonging to 16 *Vitis* species of USDA *Vitis* germplasm for the presence or absence of the *AMAT* promoter indels by genomic PCR with indel specific primers (Table S17 and Supplementary note 2). We also searched the publically available genome sequencing data of 123 *V. vinifera* cultivars, 44 *V. sylvestris* accessions, 48 *Vitis* hybrids, and 128 accessions covering 48 wild *Vitis* species for these *AMAT* structral variations^23,27,28,39–44^ (Table S18). *AMAT* alleles with absence of the 426-bp and 42-bp sequences were detected only in *V. labrusca* and its related hybrids (Tables S17 and S18). Alleles with absence of the 26-bp sequence were present in some *V. vinifera* and *V. sylvestris* accessions and also in some wild *Vitis* species. The 3-kb promoter deletion and the 2nd exon insertion, on the other hand, were found frequently, but mainly in *V. vinifera* and *V. sylvestris* accessions and the hybrids with *V. Vinifera* background (Tables S17 and S18). Interestingly, an allele with absence of the 26-bp sequence was detected in PN40024, the near-homozygous line derived from ‘Pinot noir’^18^, but not in ‘Pinot noir’ clones or Pinot-related cultivars such as ‘Pinot Meunier’, ‘Pinot blanc’ and ‘Pinot gris’ (Fig. 2 and Table S18)^40,41,44^.

**Fig. 3:**
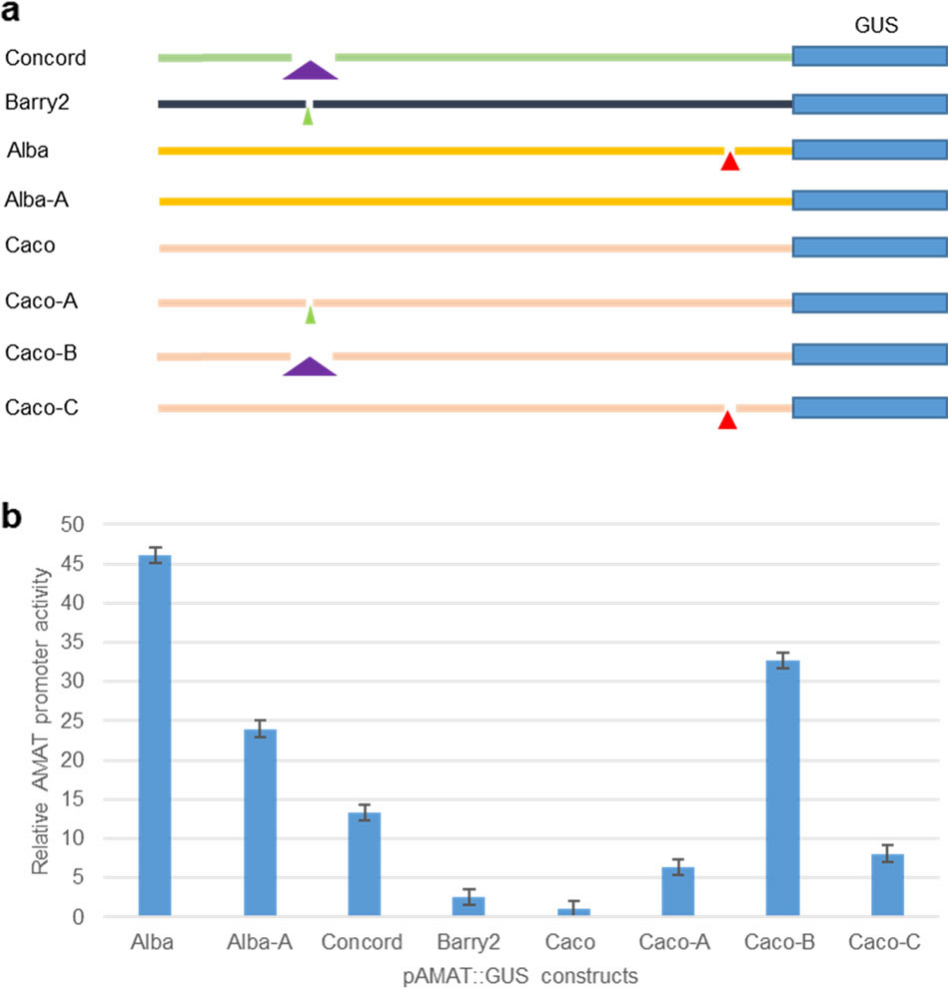
Transgenic evaluation of the impact of various indels on the *AMAT* promoter activity. **a** Four original *AMAT* promoters (2.8–3.3 kb upstream and 133 bp downstream of ATG) from ‘Concord’, ‘Barry’, ‘Alba’ and ‘Caco’ and four mutated *AMAT* promoters, Alba-A, Caco-A, Caco-B, and Caco-C, were individually cloned into a GUS reporter binary vector. These vectors covered the indel variants of 426 bp (purple triangle), 26 bp (green triangle) and 42 bp (red triangle) in different genetic background. **b** Relative *AMAT* promoter activities measured by qRT-PCR expression of the reporter *GUS* gene in transgenic *Arabidopsis* seeds. Mature seeds from 9 to 20 independent T2 lines for each construct were pooled for total RNA extraction. Two to three batches of qRT-PCR were performed for each sample with three replicates each time. Error bar is the range of fold change based on standard deviation of ΔCt value

To further validate the impact of these indels on *AMAT* expression, we evaluated promoter activities of 8 promoter variants in transgenic *Arabidopsis* seeds in light of the fact that *AMAT* was detected in grape seeds (data not shown) and the best match of *AMAT* gene in *Arabidopsis*, At5g17540, showed good expression in seeds as well (https://www.arabidopsis.org/). Eight promoter variants were tested, including four original promoters: a ‘Concord’ version, an ‘Alba’ version, a ‘Barry2’ version with absence of the 426-bp, 42-bp or 26-bp sequences, and a ‘Caco’ version with the presence of these sequences, and four artificial promoters: an ‘Alba’ mutant, ‘Alba-A’ (the 42 bp was inserted back into ‘Alba’), and three ‘Caco’ mutants, ‘Caco-A’, ‘Caco-B’ and ‘Caco-C’ with absence of the 26-bp, 426-bp, and 42-bp sequences, respectively (Fig. 3a).

Among the four original promoter variants, ‘Alba’ showed the highest promoter activity, ‘Concord’ was the second highest, and ‘Caco’ was the lowest. ‘Barry2’ had slightly higher activity than ‘Caco’, but much lower than ‘Concord’ and ‘Alba’ (Fig. 3b). These results were consistent with the *AMAT* expression data observed in these cultivars (Table S16). The three mutant ‘Caco’ promoters all had higher activities than the orginal ‘Caco’ promoter, with ‘Caco-B’ the highest and ‘Caco-A’ lowest, suggeting that elimination of the 26-bp sequence could offset some of the suppression effect of the 426-bp on *AMAT* promoter activity. On the other hand, insertion of the 42-bp sequence back into the ‘Alba’ promoter reduced its activity to about half of its original level, further supporting that the 42-bp deletion enhanced the *AMAT* promoter strength (Fig. 3b).

### *AMAT* expression and MA accumulation

MA accumulation in mature berries was assayed for 46 *Vitis* accessions. As expected, *V. labrusca* cultivars had much higher MA accumuation, with an average level of 30 or more fold higher than *V. vinifera* (Table 1). ‘Concord’ had higher MA accumualtion than ‘Catawba’ and other *Vitis* hybrids. Non-*V. labrusca* species had low MA contents (Table 1 and Table S16). These observations suggested a general correlation between *AMAT* expression and MA accumulation. Accessions with low levels of *AMAT* expression, such as ‘Caco’ and ‘Himrod’, all had low MA accumulation. However, among the accessions with high *AMAT* expression, some had low MA accumulation (Table S16). For example, ‘Hubbard’ had about 40-fold higher expression of *AMAT* than’Merlot’, but had even lower MA accumulation. ‘Catawba’ had relatively high *AMAT* expression but low MA accumulation, consistent with previous reports^11,12^ (Table 1 and Table S16). The inconsistency between *AMAT* gene expression and MA accumulation in some cultivars could be due to multiple reasons such as substrate availability or MA degradation. Nevertheless, the fact that accessions with low levels of *AMAT* expression all had low levels of MA provides convincing support that *AMAT* is the main factor responsible for MA accumulation in grape berries.

### Molecular features of *AMAT* indels

**Fig. 4:**
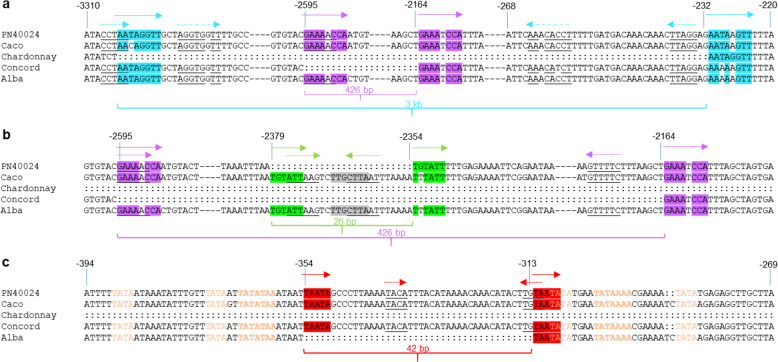
Molecular features of the major indel sites in *AMAT* promoters. DNA sequences of the *AMAT* promoters, −3310 to −220 bp upstream of the ATG start codon, from 5 different cultivars were illustrated: PN40024, Chardonnay clone I10V1, ‘Caco’, ‘Concord’ and ‘Alba’. “––” represents unspecified numbers of bases. “:” stands for absent bases. The four indels were marked by brackets of different colors: purple for the 426 bp, green for the 26 bp, red for the 42 bp, and aqua for the 3 kb. The direct repeats for each indel were highlighted and marked by solid arrows with corresponding colors. Inverted repeats (IRs) of 4–7 bp were also found for all four indels and were underlined and marked by dashed arrows facing each other. **a** features of the 3-kb indel. Two pairs of IRs were identified. **b** features of the 426-bp indel. One pair of IRs and a nested 26-bp indel were identified. The repressor binding motif (‘TTGCTTA’) of CBNAC in the 26-bp indel is highlighted in gray. **c** features of the 42-bp indel. There are six TATA elements (in orange letters) in the region, but only two of them qualified as TATA consensus sequences (TATAWAW, in bold) and the 42 bp is located between these two consensus TATA sequences

A close examination of the *AMAT* promoter indels revealed some common features (Fig. 4). They all have direct repeats (5–8 bp) at the indel boundary and inverted repeats (4–6 bp) near the indel boundaries. One inverted repeat copy at one boundary often overlaps with one direct repeat copy while the other inverted repeat copy is inside the indel boundary (Fig. 4).

A genome-wide search in both ‘Concord’ and the reference genome did not find any homologous copies of the 3-kb, 426-bp and 42-bp sequences. However, sequences highly similar to the 26-bp fragment were found at several dozen genomic sites. The 26-bp sequence contained TTGCTTA, a calmodulin-binding NAC protein (CBNAC) binding site motif which was reported to be a transcriptional repressor in *Arabidopsis*^45^, and removal of this 26-bp modestly improved the ‘Caco’ *AMAT* promoter activity (Fig. 3). The 42-bp sequence was located in a region with six TATA elements (Fig. 4). This 42 bp was part of the 56-bp sequence between the two TATA consensus sequences (TATAWAW)^46^ (Fig. 4). Deletion of the 42-bp sequence in ‘Alba’ brought these two TATA boxes into close proximity, likely contributing to the enhanced ‘Alba’ *AMAT* promoter activity. Insertion of the 42-bp sequence into the ‘Alba’ promoter reduced its activity in transgenic *Arabidopsis* seeds, confirming that spacing between these two TATA boxes is critical for the *AMAT* promoter activity (Fig. 3).

The insertions of 504 bp and 1188 bp in the 2nd exon were located at the same position. Both had the same 5-bp direct repeats, likely a target site duplication (TSD) sequence (Fig. 2 and Supplementary note 7, Fig. S2). The larger insertion is a terminal-repeat retrotransposon in miniature (TRIM)^47^ with 5′LTR (long terminal repeat) of 499 bp, an internal region of 188 bp with a primer binding site and a polypurine tract motif, and 3′LTR (496 bp). By contrast, the smaller insertion is a solo LTR of 499 bp. This suggests that the soloLTR which was located at the same position was likely generated through a recombination between the two LTRs in the complete TRIM (Supplementary note 7, Fig. S2). A blast with the 499-bp soloLTR identified hundreds of copies across all the 19 chromosomes in the PN40024 genome (Table S19). Furthermore, a cluster of 6 LTRs were found on Chromosome 6, with a 190 bp sequence between the elements (Table S19). This LTR seems specific to *Vitis* and no similar sequences were found in other plant species.

We examined some RNA-Seq data for the impact of this exonic insertion on *AMAT* gene expression. Besides TRIM insertion, *VT* alleles (*V. vinifera AMAT* allele with the TRIM insertion) had additional SNPs to distinguish them from other *AMAT* alleles (Supplementary note 7, Fig. S2 and Table S14). In the case of ‘Xiangfei’ which had a TRIM insertion (Fig. 1 and Table S18), all the RNA-Seq reads mapped to the TRIM insertion site in the exon 2 region had a 4-bp frameshift insertion at position 597 (Supplementary note 7, Fig. S2 and Table S14), suggesting that ‘Xiangfei’ had only *VT* alleles. *AMAT* of ‘Xiangfei’ was expressed at a very low level, with TPM value less than 15 while other three *V. vinifera* cultivar had values more than 200 at ripening stage (Fig. 1), supporting that the large exonic TRIM insertion negatively impacts *AMAT* expression.

## Discussion

‘Foxy’ aroma is a complex trait and there are at least three known contributing chemicals: MA, 2-Aminoacetophenone (2-AAP) and furaneol^9,12,13,15^. We focused on the genetic control of MA biosynthesis in this study because MA is the predominant compound for ‘foxy’ aroma and two genes (AMAT and AAMT) responsible for the last step of MA biosynthesis have been identified^13,16,17^. We found *AMAT*, one *AAMT1-*like, and a few *AMAT-*like genes differentially expressed at berry veraison and ripening stages between ‘Concord’ and *V. vinifera* (Tables S12 and S13). We also examined other known genes in pathways that might be related to the ‘foxy’ aroma, including those for biosynthesis of 2-AAP and furaneol, and none were differentially expressed between ‘Concord’ and *V. vinifera* at the ripening stage (Supplementary note 3 and Supplementary Table 13). These results support the long-known fact that MA was the dominant ‘foxy’ compound in *V. labrusca*, even though not all *V. labrusca* related grapes accumulated high levels of MA^11,12,15,48^.

While both *AMAT* and *AAMT1* were differentially expressed at veraison and ripening stages and showed many folds higher expression in ‘Concord’ than in *V. vinifera* (Fig. 1 and Tables S12 and S13), *AMAT* appeared to be the major contributor to MA accumulation in ‘Concord’ and most other *V. labrusca*-derived cultivars. In general, *AMAT* had much higher expression than *AAMT1* and MA accumulation was much more closely correlated with the expression levels of *AMAT* across various germplasm accessions (Table S16). As an extreme example for supporting this conclusion, the hybrid ‘Steuben’ had essentially no expression of *AMAT* and no accumulation of MA, although it had a high level of *AAMT1* expression (Table S13). Nevertheless, the role of *AAMT1* contributing to MA synthesis is apparent in some genetic backgrounds. For example, ‘Niagara’ had very high MA accumulation and high *AAMT1* expression, but relatively low *AMAT* expression (Tables S13 and S16)^12^. Recently, an *AAMT1* gene was identified as a potential candidate underlying a QTL of MA content in ‘Catawba’^49^, also suggesting its important role in MA biosynthesis. The quantitative nature and multiple-gene control of MA content were previously proposed from studies of grapevine segregation populations^50,51^.

We revealed several promoter indels responsible for differential *AMAT* expression among different species and genotypes in this study. While the deletion of the entire 3-kb promoter region, the TRIM insertion in exon 2, and even the loss of the whole locus were dramatic changes responsible for no or much reduced *AMAT* expression in some accessions with *V. vinifera*- and *V. sylvestris*-pedigrees, the 426-bp and 42-bp promoter indels were the main, common causes for differential *AMAT* expression between *V. labrusca* and non-*V. labrusca* grapes. Presence of direct and inverted repeats and their similar distribution patterns found in the 3-kb, 426-bp, 26-bp and 42-bp indels (Fig. 4) suggest a potential common mechanism for how these indel sequences might be generated. Among various mechanisms, homology-based replication slippage^52–55^ might be a possible cause for generating these *AMAT* indels. The simultaneous presence of both direct and inverted repeat pairs could lead further various types of structural changes in these indels.

‘Foxy’ aroma is a unique attribute of ‘Concord’ and other grapes with *V. labrusca* parentages and represents a special trait associated with the culture of early settlers. Here we elucidated the genetic control of *AMAT* and provided a step closer to fully dissecting this important aroma trait. The discovered indels responsible for the differential expression of *AMAT* between *V. labrusca* and *V. vinifera* grapes provide useful molecular markers for tracking and selecting for or against ‘foxy’ aroma in grapevine breeding. Likewise, the draft ‘Concord’ genome offers unique, valuable resources for gene discovery and trait improvement in juice grapes.

## Methods

### Plant material, DNA and RNA isolation

Plant materials used in this study were listed in Table 1, Tables S16, S17, and S20. All samples were frozen in liquid nitrogen and stored in −80 °C for further processing. High molecule weight genomic DNA for construction of mate-pair libraries was prepared using illustra DNA Extraction kit PHYTOPURE (GE Healthcare). DNA for all other analyses was isolated using Qiagen DNAeasy Plant Kit. RNA was extracted using the Spectrum Plant Total RNA Kit (Sigma-Aldrich, Kansas City, MO, USA).

### Genomic and RNA-Seq library construction and sequencing

See Supplementary note 4.

### Genome size, heterozygosity and assembly

See Supplementary note 4.

### Construction of collinear blocks

See Supplementary note 4.

### Repeat annotation, gene prediction and functional annotation

See Supplementary note 4.

### RNA-Seq data analysis

See Supplementary note 4.

### Expression analysis of the *AMAT* gene

Mature berry samples were collected in September 2015 and a subset of the samples was also collected in September 2016. Total RNA from berry skin was first treated with Turbo DNA-free kit to remove genomic DNA (Thermo Fisher Scientific). Messenger RNAs (mRNAs) were enriched using oligo-dT magnetic beads (New England Biolabs). cDNAs were reverse-transcribed (RT) from purified mRNAs using oligo(dT)_18_ primers by Revert Aid first strand cDNA synthesis kit (Thermo Fisher Scientific). Two sets of *AMAT*-specific primers (c5F/c5R at 5′ end and c4F/c1R at 3′ end) were used for qRT-PCRs (Table S21). *EFa1* (EC959059) was used as an internal control (Table S21). Three replicates of each sample and a negative control (water) were analyzed. Relative expression was assessed using the comparative ΔΔCt method^56^. Similar results were obtained for both sets of *AMAT* primers and the c4F/c1R results were presented in Table 1 and Table S16.

### *AMAT* genomic DNA and cDNA cloning

*AMAT* DNA sequences were PCR-amplified from genomic DNA using the outF/outR primer pair (Table S21). *AMAT* cDNAs (ORF) were obtained by RT-PCR amplification of berry skin RNA samples using the c5F/c7R primer pair (Table S21). PCR products were cloned into pCR®8/GW/TOPO® vector (Thermo Fisher Scientific). The whole insert was sequenced with vector primers and gene-specific primers.

### Construction of *AMAT* promoter-GUS reporter binary vectors

The roles of certain indels in affecting *AMAT* promoter activities were investigated in transgenic *Arabidopsis* seeds using *beta-glucuronidase* (*gus-A*) as a reporter gene. The *AMAT* promoter sequence variants of interest were respectively amplified from the genomic DNA of grape cultivars ‘Concord’, ‘Barry’, ‘Caco’, and ‘Alba’, using primers OutF and c5R (Fig. 3 and Table S21) which amplified a 3–3.4 kb *AMAT* genomic region, including the 2845 bp to 3275 bp sequence before and a 133 bp after the ATG start codon. The 3–3.4 kb PCR product was cloned into a pCR8/GW/Topo vector (Thermo Fisher Scientific). The promoter length for ‘Alba’, ‘Caco’, ‘Barry2’ (the longer allele in ‘Barry’ which lacked the 26-bp indel) and ‘Concord’, is 3408 bp, 3405 bp, 3367 bp and 2978 bp, respectively. These *AMAT* promoter sequence variants were cloned into the *GUS* reporter gateway binary vector *pGWB533*^57^. Four *AMAT* promoter mutants were also generated using the ‘Caco’ or ‘Alba’ construct as backbone. For the mutant engineering, the original *pGWB533* vector was double digested by HindIII and MfeI to release the entire gateway cloning cassette and 135-bp GUS coding sequence. Primer 1215 F (5′-GTAAAACGACGGCCAGTGCCAAGCTTG-3′ (covering the HindIII site in pGWB533) and 1216 R (5′- GTTAAAACTGCCTGGCACAGCAATTGC-3′, covering the MfeI site in the GUS coding region in *pGWB533*) were used with other primers (Table S21) to amplify different segments of the *AMAT* promoter sequence in different *pGWB533*-*AMAT* constructs. The mutant construct was assembled with the *pGWB533*-HindIII/MfeI and various overlapping *AMAT* fragments using NE Builder HiFi DNA Assembly cloning kit (New England Biolabs). The entire 3.4-kb *AMAT* promoter region for individual constructs was sequenced to confirm that the intended mutations were introduced without any extra mutations.

Verified constructs were transformed into *Arabidopsis* via *Agrobacterium* using the floral dipping method^58^. Genomic PCR with primers located on the *AMAT* promoter was used to confirm the insertion of the *pAMAT-GUS* cassette in each transgenic *Arabidopsis* T1 line. T2 seeds were collected from individual transgenic plants. Mature seeds from 9–20 lines for each construct were pooled and stored in −80 °C until RNA extraction.

Seeds were ground in liquid nitrogen together with 50 mg sand using mortar and pestle. RNA extraction and reverse transcription were similar as described in the “Expression analysis of the *AMAT* gene”. 0.5 µl cDNA was used for each quantitative PCR reaction in 20 μl volume. The *Actin* gene, *At3g18780*, was used as the control (primers 152 F/170 R, Table S21). The expression of the *GUS* gene (primers 1207 F/49 R, Table S21) was used as the reporter to quantify the *AMAT* promoter activities of different constructs. Two to three batches of qRT-PCR were performed for each sample with three replicates each time.

### Germplasm survey for structural variation at the *AMAT* locus

Primers crossing the indel junction (426 bp, 42 bp, and 26 bp) (Table S21) were paired with primers in 200–500 bp distance on the *AMAT* promoter to check the indel status (positive result means the indel is absent in at least one allele). Primers inside the indel were paired with primers in 200–500 bp distance on the *AMAT* promoter to check the presence of the indel (positive result means the indel is present in at least one allele). The 3-kb promoter deletion was revealed by shorter PCR product with primers outside the 3-kb indel. The exon 2 TRIM insertion was examined by LTR-specific primer and exon 2 primer. However, the genomic PCRs often had background of multiple PCR bands for TRIM detection indicating the complication of this type of insertion, likely due to presence of the LTRs in opposite directions next to each other at some genomic loci (Table S19).

### Survey for structural variations at the *AMAT* locus with public genome sequences

To survey the public genomic resources for *AMAT* structural variations, we used template sequences specific to different indel boundaries (Supplementary note 5) to blast the SRA files of *V. vinifera* and many *Vitis* species (Table S18). For detection of alleles with absence of the 26-bp, 42-bp, 426-bp or 3-kb sequence, query templates consisting of a 160-bp sequence crossing the indel boundaries with 80 bp from each side of the indel were used for read alignments. For the TRIM insertion in the 2nd exon as found in ‘Pinot noir’ ENTAV 115 and ‘Chardonnay’, two templates were used, one consisting of 80 bp from the left insertion boundary of the exon 2 and 80 bp from the 5′ end of the solo LTR, the other consisting of 80 bp from the 3′ end of the solo LTR element and 80 bp from the right insertion boundary of the exon 2. Accessions with reads crossing the junction region (especially the 60–100 bp in the template) would suggest that these accessions contained alleles with the specific deletion or insertion. The two templates used for the exon 2 insertion survey most often had similar results (the accessions with reads crossing one template will also had reads crossing the other template), but with some exceptions (only one template had crossing reads but not the other), likely due to low genomic coverage. It should be noted that use of these two templates for the exon 2 insertion survey would not be able to determine if an insertion had a solo LTR (504 bp) or a full-length TRIM (1188 bp) since both cases would have similar junction sequences.

### MA extraction and gas chromatography

About 60–80 representative berries from two vines were collected as bulk in the 2018 season from the *Vitis* collection in Geneva, NY at maturity, frozen and stored at −20 °C. Approximately 100–200 ml of juice were extracted from thawed berries, and 5 ml of juice were added to 4 ml of water and 1 ml of 10 μg ml^−1^ 2-octanone standard, with each accession prepared in triplicate. Samples were mixed and centrifuged for 10 min at 6,000 g. Methyl anthranilate was extracted using solid-phase extraction with 3 ml 200 mg LiChrolut EN 3 tubes connected to a positive pressure manifold under nitrogen gas and ethyl acetate as solvent. Columns were preconditioned with 4 ml of ethyl acetate, methanol, and finally, model juice with pH 3.5 (50 g L^−1^ fructose, 50 g L^−1^ glucose, and 6 g L^−1^ tartaric acid) at a rate of 2–3 ml min^−1^. A total of 10 ml of juice were passed through column at 1 ml min^−1^, followed by 750 μl of water, and dried under nitrogen gas. MA was eluted using 1.5 ml of ethyl acetate.

Separation and analysis were performed on an Agilent 7890 A GC coupled with Agilent 5977E mass selective detector. Data acquisition and processing was done using OpenLab software (Agilent). Chromatographic conditions were in split less mode at a flow rate of 15 ml min^−1^ at 200 ^o^C using 5.0 purity helium carrier^15^. A CP-WAX 52 CB (30 m × 0.25 mm × 0.25 μm) capillary column (Varian, Lake forest, CA, USA) at a flow rate of 0.5 ml min^−1^ was used for separation. Initial oven temperature was held at 60 ^o^C for 1 min and increased at 10 ^o^C per min to 200 ^o^C, held for 5 min, then increased to 220 ^o^C at 10 ^o^C per min. Detector temperature was 200 ^o^C. MA was detected in Selected Ion Monitoring mode for specific ions at 92, 119, and 151 (m/z). Standard curve for MA was established using standard from Sigma.

## Supplementary information

SupplementaryNotes3.19.2020Clean

AMATSupplementaryTables3.18.2020Final

## Data Availability

The Concord genome assembly and raw genome sequencing reads have been deposited in the NCBI BioProject database (http://www.ncbi.nlm.nih.gov/bioproject) under the accession number PRJNA606024. Raw genome resequencing and transcriptome sequencing reads have been deposited under accession numbers PRJNA606045, PRJNA606274, and PRJNA606742.
