# Supplementary material for: A key ‘foxy’ aroma gene is regulated by homology-induced promoter indels in the iconic juice grape ‘Concord’
Source: Hortic Res. 2020 Apr 18;7:67. doi: 10.1038/s41438-020-0304-6 (PMC7166211; doi:10.1038/s41438-020-0304-6)
Supplement: Supplementary file 1 — SupplementaryNotes3.19.2020Clean [file 41438_2020_304_MOESM1_ESM.docx]

**A key ‘foxy’ aroma gene is regulated by homology-induced promoter indels in the iconic juice grape ‘Concord’**

Yingzhen Yang^1^, José Cuenca^1,2^, Nian Wang^1,3^, Zhenchang Liang^4^, Honghe Sun^5^, Benjamin Gutierrez^6^, Xiaojun Xi^6,7^, Jie Arro^6^, Yi Wang^4,8^, Peige Fan^4^, Jason Londo^1^, Peter Cousins^9^, Shaohua Li^4^, Zhangjun Fei^5,10^ & Gan-Yuan Zhong^1*^

^1^ US Department of Agriculture-Agricultural Research Service, Grape Genetics Research Unit, Geneva, New York, USA.

^2^ Present address: Centro de Citricultura y Producción Vegetal. Instituto Valenciano de Investigaciones Agrarias, Moncada, Valencia, Spain.

^3^ Present address: College of Horticulture and Forestry, Huazhong Agricultural University, Wuhan, Hubei, China.

^4^ Beijing Key Laboratory of Grape Sciences and Enology, Laboratory of Plant Resources, Institute of Botany, Chinese Academy of Sciences, Beijing, China.

^5^ Boyce Thompson Institute for Plant Research, Cornell University, Ithaca, NY, USA.

^6^ US Department of Agriculture-Agricultural Research Service, Plant Genetic Resources Unit, Geneva, New York, USA.

^7^ Forestry and Pomology Research Institute, Shanghai Academy of Agricultural Sciences, Shanghai, China.

^8^ University of Chinese Academy of Sciences, Beijing, China

^9^ E. & J. Gallo Winery, Modesto, CA, USA.

^10^ US Department of Agriculture–Agricultural Research Service, Robert W. Holley Center for Agriculture and Health, Ithaca, NY, USA

^*^ Correspondence: ganyuan.zhong@ars.usda.gov

These authors contributed equally: Yingzhen Yang, José Cuenca, Nian Wang, Zhenchang Liang

**Contents**

**1. Supplementary Note 1: ‘Concord’ genome sequence, assembly and pedigree**

**2. Supplementary Note 2: Pedigree information from NPGS GRIN-Global**

**3. Supplementary Note 3: Pathways and genes related to ‘foxy’ aroma**

**4. Supplementary Note 4: Supplementary Methods**

**5. Supplementary Note 5: Blasting templates for detection of indels in *AMAT* genomic region**

**6. Supplementary Note 6: References**

**7. Supplementary Note 7: Supplementary figures**

**8. Supplementary Note 8: List of supplementary tables**

**Supplementary Note 1**

**‘Concord’ genome sequence, assembly and pedigree:**

We used a whole-genome shotgun strategy for ‘Concord’ genome sequencing and assembly. A total of 108 Gb from 423 million cleaned Illumina paired-end reads were generated by sequencing DNA libraries with the fragment sizes of 260 bp, 500 bp, 5 kb, 10 kb and 20 kb (Table S1). This represents 216-fold coverage of the ‘Concord’ genome. Based on the frequency distribution of 21-mers (Figure S1), ‘Concord’ genome was estimated to be 499 Mb, similar to the *V. vinifera* ‘Pinot noir’ genome ^1,2^. *De novo* assembly of the Illumina reads, however, resulted in a final assembly of 570.8 Mb ‘Concord’ genome, which is ca. 70Mb larger than the reference ‘Pinot noir’ genome ^1–3^. The assembly consisted of 46,367 contigs (length >200 bp) (N_50_ = 20.3 kb) and 9,829 scaffolds (N_50_= 465.7 kb) of which the longest contig and scaffold length were 217.3 kb and 4.91 Mb, respectively (Table S2).These assembly statistics can certainly be much improved by employing some of the most contemporary sequencing and assembly techniques, such as the PacBio^TM^ platform, the Oxford Nanopore MinION^TM^ technology and the optical mapping technology ^4–10^.

We assessed the quality of the ‘Concord’ assembly by using several independent data sources. A total of 28 ‘Concord’ RNA-Seq libraries were generated from various tissues (Table S3). Short reads from these 28 RNA-Seq libraries were mapped to the assembled ‘Concord’ scaffolds, which revealed high mapping rates, ranging from 79.2% to 88.7% with an average of 84.0% (Table S3). The completeness of the ‘Concord’ assembly was further assessed with BUSCO v3 and the embryophyta_odb10 database ^11^, which showed that 93.2% of the core conserved plant genes (a total of 1,375) were completely covered by the assembly (91.6% were covered once and 1.6% covered twice or more), and another 3.3% were partially covered, while only 3.5% were missing in the ‘Concord’ genome assembly. Together, these results suggested that the ‘Concord’ genome assembly captured the majority of the gene spaces.

A genome-wide comparison between ‘Concord’ and *V. vinifera* PN40024 genomes revealed that 54.1% of the PN40024 genome (254 Mb) was represented in 25,744 collinear blocks (> 5kb) in ‘Concord’ (Table S4). Around 44.4% (ca. 254 Mb) of the assembly were identified as repetitive sequences, which was lower than 48.3% for the PN40024 genome and assessed using the same parameters. However, examination of different categories of repetitive sequences, such as SINEs, LINEs, and LTR elements, revealed similar content between the ‘Concord’ assembly and the PN40024 genome (Table S5). A total of 25,499 protein-coding genes were predicted in the ‘Concord’ genome (Table S6). The predicted genes had an average genomic and coding sequence length of 5,591 and 1,336 bp, respectively (Table S7), and an average of 5.2 exons with a mean exon length of 260 bp. Functional annotation confirmed that 98.5% of the predicted genes had known homologues in public protein databases, with 21,440 homologues in database InterPro, 16,052 in GO, 20,419 in Swissprot and 25,060 in TrEMBL (Table S8).

To provide a genome-wide assessment of ‘Concord’ pedigree, we generated 170.4 Gb of short sequence reads for *V. vinifera* ‘Semillon’, hybrid grapes ‘Catawba’ and ‘Concord’, four *V. labrusca* accessions which shared most SSR marker profiles with ‘Concord’ (https://npgsweb.ars-grin.gov) and one *V. amurensis* accession as an out group (Table S10). Raw reads were processed to remove duplicates and adaptor and low-quality sequences using Trimmomatic ^12^. The cleaned reads were aligned to the Concord or PN40024 genome using BWA-MEM ^13^. SNPs were then called using GATK ^14^ following the online Best Practices protocol with recommended parameters (<https://software.broadinstitute.org/gatk/best-practices/>). To predict the pedigree of ‘Concord’, the allele of ‘Concord’ at each SNP position was compared with the allele in each of the seven other accessions. The number of shared alleles between ‘Concord’ and each of the seven accessions at all SNP loci was derived. Based on genotypic concordance of about 13 million SNPs, ‘Catawba’ showed the highest possibility to be a parent of ‘Concord’ (97.9% with ‘Concord’ as the reference) (Table S10). ‘Wheeler’ showed the highest concordance with ‘Concord’ (95.3% with ‘Concord’ as the reference) among the four *V. labrusca* accessions. ‘Semillon’ shared a decent level (72.9%) of SNP concordance with ‘Concord’. These results provide genome-wide evidence supporting the pedigree relationships among ‘Concord’, ‘Catawba’ and ‘Semillon’ previously drawn based on the SSR data ^15^.

**Supplementary Note 2**

**Pedigree information from NPGS GRIN-Global**

Most of the germplasm accessions used in this study were from the USDA Agricultural Research Service National Plant Germplasm System (NPGS). The pedigree information provided in the NPGS GRIN-Global (<https://npgsweb.ars-grin.gov>) for some of the *Vitis* accessions might not be accurate ^16^. This is a typical problem for many germplasm repositories in which some genetically identical accessions were given different names and, likewise, genetically different accessions were given the same names due to many historical reasons. We had such a case in this study. Hybrids ‘Ulster’ and ‘Clinton’ accessions (Table S16) showed the same microsatellite profiles (<https://npgsweb.ars-grin.gov>) and they most likely represent the same hybrid. Indeed, we found that these two accessions had the identical *AMAT* indel genotypes and their relative *AMAT* expression and MA accumulation levels were very similar (Table S16). Similarly, the identities for some of the *V. labrusca* accessions from GRIN-Global might be questionable and instead they might be hybrids between *V. labrusca* and *V. vinifera*. There is ongoing effort in the NPGS to resolve the identity issues of *Vitis* collection. This issue, however, should not present a problem for the current study, as the analyses and conclusions were drawn based on the *AMAT* indel genotypes.

**Supplementary Note 3**

**Pathways and genes related to ‘foxy’ aroma**

‘Foxy’ aroma is a complex trait and there are at least three known contributing chemicals: MA, 2-Aminoacetophenone (2-AAP) and furaneol^17–20^. Very little is known about genes responsible for 2-AAP biosynthetic pathways ([www.metacyc.org](http://www.metacyc.org))^21^. Recently several genes have been suggested to play a role in furaneol synthesis in strawberry, including quinone oxidoreductase (FaQR)^22^, O-methyltransferase (FaOMT)^23,24^, an ethylene response factor and a MYB gene^25^. However, grape counterparts of these genes did not show much expression difference between ‘Concord’ and *V. vinifera* berries (Table S13). Additionally, a grape UDP-glucose:furaneol glucosyltransferase gene (Vitvi07g01394) was suggested to involve in the biosynthesis of furaneol^26^, but this gene was actually expressed significantly higher in *V. vinifera* than in ‘Concord’ at the veraison stage though not at the ripening stage (Tables S12 and S13).

**Supplementary Note 4**

**Supplementary Methods**

**Genomic and RNA-Seq library construction and sequencing**

For ‘Concord’ genome assembly, four paired-end libraries (Table S1), one with insert size of 260 bp and three with insert sizes of 500 bp, were prepared using the Genomic DNA Sample Prep kit (Illumina, CA) or the DNA Library Preparation kit (Kapa Biosystems, MA) according to the manufacturers’ protocols. Three mate‐pair libraries with insert sizes of 5, 10 and 20 kb, respectively, were prepared with the Nextera Mate Pair Sample Preparation kit (Illumina, CA). For genome resequencing, paired-end DNA libraries were constructed for *V. vinifera* ‘Semillon’, *Vitis* hybrid ‘Catawba’, four *V. labrusca* accessions and one *V. amurensis* accession (Table S10) using the Genomic DNA Sample Prep kit (Illumina, CA). For transcriptome profiling, RNA-Seq libraries were constructed according to the published protocols ^27,28^. All DNA and RNA-Seq libraries were sequenced on a HiSeq 2500 system with the paired-end mode.

**Genome size, heterozygosity and assembly**

Illumina read pairs from the libraries involving PCR amplification were processed with the package of “Fastuniq” ^29^ to remove PCR-generated duplicated reads. Read processing including removal of adaptor and low-quality sequences was performed by Trimmomatic ^30^. To estimate the genome size and heterozygosity of ‘Concord’, k-mer distribution (k=21) was calculated using jellyfish ^31^ on the basis of all cleaned reads from paired-end libraries. The genome size was predicted using a k-mer counting approach ^32^.

Cleaned ‘Concord’ paired-end and mate-pair reads were subjected to error correction using the ALLPATHS-LGpackage ^33^ and then assembled into scaffolds using Platanus which can effectively manage high-throughput data from heterozygous samples ^34^. We improved the de novo assembly by aligning all scaffolds (length >2 kb) to the PN40024 genome to detect large structural variation within collinear blocks (gaps or translocation larger than 5 kb) in the ‘Concord’ scaffolds and then re-mapping all the short reads which were used for assembling and scaffolding onto ‘Concord’ scaffolds.

**Construction of collinear blocks**

Pairwise whole genome comparisons were conducted by aligning the assembled scaffolds of ‘Concord’ to the PN40024 genome using the NUCmer program ^35^. The alignment results were further filtered to retain one-to-one alignment regions using the “delta-filter” program implemented in MUMmer packages ^35^.

**Repeat annotation, gene prediction and functional annotation**

An approach combining de novo and homology was employed for genome-wide repeat sequence annotation. De novo repeat libraries from the genome sequences of ‘Concord’ and the *V. vinifera* reference genome PN40024 (version 2 from <http://www.phytozome.com/>) were constructed using LTR_FINDER, PILER and RepeatScout with default parameters ^36–38^. After consensus sequence and classification information for each repeat family were constructed, RepeatMasker was applied for searching homology repeat sequence (http://www.repeatmasker.org).

MAKER (2.31.6) was applied for predicting protein-coding genes in the ‘Concord’ genome using the integrated evidence from *ab initio* gene prediction, transcript mapping and protein homology ^39^. AUGUSTUS and SNAP were used for *ab initio* gene predictions ^40,41^. To generate transcript sequences, cleaned reads produced from the 28 RNA-Seq libraries (Table S3) were de novo assembled using Trinity ^42^. Reads from different biological replications of the same tissue were assembled together. Then the CD-HIT package ^43^ (v4.6.1-2012-08-27) was applied to cluster transcripts produced from different tissues, with a sequence identity cutoff of 0.95. Through these filtering and processing, a total of 450,020 ‘Concord’ transcripts were obtained for further gene prediction. More than 300,000 *V. vinifera* ESTs publicly available from GeneBank were used as additional transcript evidence during the MAKER prediction. In addition, protein sequences from the Swiss-Prot database were used to provide protein homology evidence.

To perform gene functional annotation, a local “lookup_service” database and InterProScan version5 package ^44^ were employed. Meanwhile, all ‘Concord’ predicted gene sequences were blasted to the UniProtprotein database (Swiss-Prot/TrEMBL) ^45^. An E value cutoff of 1e-4 was used for both blast and InterProScan.

**RNA-Seq data analysis**

Quality control and adaptor removing for the raw RNA-Seq reads were performed by Trimmomatic^30^. The resulting cleaned reads from *V. vinifera* ‘Merlot’, ‘Jingzaojing’, ‘Xiangfei’ and ‘Jingxiu’ as well as ‘Concord’ were mapped to the genome of *V. vinifera* PN40024 (version 2) by tophat2^46^. Differential expression analyses were performed using the Cufflinks package, following the Tuxedo protocol^47^. The count mapped reads for each gene extracted from the aligned reads using HTseq^48^ and final expression unit per gene were reported in TPM (transcript per million reads mapped).

**Supplementary Note 5**

**Blasting templates for detection of indels in *AMAT* genomic region** (left border sequence underlined and left border sequence in italic)

Template for **3-kb promoter deletion** as in Chardonnay clone I10V1 000028F contig QGNW01000024.1

TAAAATTGTGCATCCTTTTATCATATTTAGTAGATTATTTAGTGGTTGAGAGATCACCCATTGTTTTTTATATCTAATAG*GTTTTTACCTTAAGAGAAAAATACAAGTGAAGCACACTTAAAGTTCTACCTCTCATCTACACCGTGTCATAGGCTGAGCA*

Template for **426-bp deletion** as in ‘Concord’

ACTTATTTTTATTTTCTTTGAATTAGTTTGAAACCTCGTTTCAAAATCCAATTTTTTTAAATAAGGTTCTAAATGTGTAC*GAAATCCATTTAGCTAGTGACAACATTTACAAAAGATAGTAAAACTATTAAGGGAGTTACGATGAGCATTCAAGGGCTTG*

Template for **26-bp deletion** as in PN40024

CGGGAAATTTTGACATTAATTGGCTTGATTTTTTAATTTGAAAATAATTTTAAAAACTATGAAACTAATATTAAATTTAA*TGTATTTTTGAGAAAATTCAGAATAACATAATCATTTTTAGAGAAGAAATTAATATAAACATTCTATACCAATTTCAATT*

Template for **42-bp deletion** as in ‘Alba’

TCACTTTTTAATAATTCCTAAATATTCCCCCCATTATTTTTATAATAAATATTTGTTTATAATTATATAAATAATTAATA*TATGAATATAAAACGAAAATCTATAAGAGAGGTTGCTTAATTCAAACACCTTTTTGATGACAAACAAACTTAGGAGAAAA*

Template for **TRIM insertion** in Exon 2 (left boarder of Exon 2 (underlined) plus 5’LTR (bold)) as in Chardonnay clone I10V1 000028F contig QGNW01001232

TACTAAGGCTGGAATGCTATGTAAAAATCCATTGGAATTTGCGATAAGGTTAGTGAAGAAAGCCAAGGTGGAAATGAGCC**AGGAGTGTTGGGAATTGTCCCAAATTCCTAATTAGTAAATATTCCTTTTTGTAAAGAATATTGTATAGAATATTTCTAGG**

Template for **TRIM insertion** in Exon 2 (3’LTR (bold) plus right boarder of Exon 2 (italic)) as in Chardonnay clone I10V1 000028F contig QGNW01001232

**GTGTACCGTATGTGTGATTGTTTTTATCTTTGTGTTCTTGAACTAACATCGTTGGCATCAAAGCTTCCGCTGGCACAACA***AGGAGTACATTAAATCAGTGGCAGATCTTATGGTCATCAAGGGCCGGCCCTCATTTACGCAGCCAGGGAACTATATTGTT*

**Supplementary Note 6**

**References**

1. Jaillon, O. *et al.* The grapevine genome sequence suggests ancestral hexaploidization in major angiosperm phyla. *Nature* **449**, 463–467 (2007).

2. Velasco, R. *et al.* A high quality draft consensus sequence of the genome of a heterozygous grapevine variety. *PLoS One* **2**, e1236 (2007).

3. Canaguier, A. *et al.* A new version of the grapevine reference genome assembly (12X.v2) and of its annotation (VCost.v3). *Genomics Data* **14**, 56–62 (2017).

4. Chin, C. S. *et al.* Phased diploid genome assembly with single-molecule real-time sequencing. *Nat. Methods* **13**, 1050–1054 (2016).

5. Teh, B. T. *et al.* The draft genome of tropical fruit durian (Durio zibethinus). *Nat. Genet.* **49**, 1633–1641 (2017).

6. Lu, H., Giordano, F. & Ning, Z. Oxford Nanopore MinION Sequencing and Genome Assembly. *Genomics, Proteomics and Bioinformatics* vol. 14 265–279 (2016).

7. Jain, M. *et al.* Nanopore sequencing and assembly of a human genome with ultra-long reads. *Nat. Biotechnol.* **36**, 338–345 (2018).

8. Daccord, N. *et al.* High-quality de novo assembly of the apple genome and methylome dynamics of early fruit development. *Nat. Genet.* **49**, 1099–1106 (2017).

9. Ansorge, W. Next Generation DNA Sequencing (II): Techniques, Applications. *J. Next Gener. Seq. Appl.* **01**, (2016).

10. Miller, D. E., Staber, C., Zeitlinger, J. & Hawley, R. S. Highly Contiguous Genome Assemblies of 15 Drosophila Species Generated Using Nanopore Sequencing . *G3&amp;#58; Genes|Genomes|Genetics* **8**, 3131–3141 (2018).

11. Simão, F. A., Waterhouse, R. M., Ioannidis, P., Kriventseva, E. V. & Zdobnov, E. M. BUSCO: Assessing genome assembly and annotation completeness with single-copy orthologs. *Bioinformatics* (2015) doi:10.1093/bioinformatics/btv351.

12. Blanco-Ulate, B. *et al.* Red blotch disease alters grape berry development and metabolism by interfering with the transcriptional and hormonal regulation of ripening. *J. Exp. Bot.* **68**, 1225–1238 (2017).

13. Li, H. Aligning sequence reads, clone sequences and assembly contigs with BWA-MEM. *arXiv Prepr. arXiv* (2013) doi:arXiv:1303.3997 [q-bio.GN].

14. McKenna, A. *et al.* The genome analysis toolkit: A MapReduce framework for analyzing next-generation DNA sequencing data. *Genome Res.* **20**, 1297–1303 (2010).

15. Huber, F. *et al.* A view into American grapevine history: Vitis vinifera cv. ‘Sémillon’ is an ancestor of ‘Catawba’ and ‘Concord’. *Vitis - J. Grapevine Res.* **55**, 53–56 (2016).

16. Sawler, J. *et al.* Genomics assisted ancestry deconvolution in grape. *PLoS One* **8**, 1–8 (2013).

17. Sun, Q., Gates, M. J., Lavin, E. H., Acree, T. E. & Sacks, G. L. Comparison of odor-active compounds in grapes and wines from vitis vinifera and non-foxy American grape species. *J. Agric. Food Chem.* **59**, 10657–10664 (2011).

18. Acree, T. E., Lavin, E. H., Nishida, R. & Watanabe, S. O-Amino acetophenone the ‘foxy’ smelling component of labruscana grapes. in *Flavour Science and Technology - 6th Weurmann Symposium* (eds. Bessiere, Y. & Thomas, A. F.) 49–52 (Wiley-Blackwell, 1990).

19. Wang, J. & Luca, V. De. The biosynthesis and regulation of biosynthesis of Concord grape fruit esters, including ‘foxy’ methylanthranilate. *Plant J.* **44**, 606–619 (2005).

20. Prudêncio Dutra, M. da C. *et al.* Rapid determination of the aromatic compounds methyl-anthranilate, 2′-aminoacetophenone and furaneol by GC-MS: Method validation and characterization of grape derivatives. *Food Res. Int.* **107**, 613–618 (2018).

21. Frydman, R. B. ., Tomaro, M. L. . & Frydman, B. Pyrrolooxygenases: the biosynthesis of 2-aminoacetophenone. *FEBS Lett.* **17**, 273–276 (1971).

22. Raab, T. FaQR, Required for the Biosynthesis of the Strawberry Flavor Compound 4-Hydroxy-2,5-Dimethyl-3(2H)-Furanone, Encodes an Enone Oxidoreductase. *Plant Cell Online* **18**, 1023–1037 (2006).

23. Wein, M. *et al.* Isolation, cloning and expression of a multifunctional O-methyltransferase capable of forming 2,5-dimethyl-4-methoxy-3(2H)-furanone, one of the key aroma compounds in strawberry fruits. *Plant J.* **31**, 755–765 (2002).

24. Lunkenbein, S. *et al.* Up- and down-regulation of Fragaria×ananassa O-methyltransferase: impacts on furanone and phenylpropanoid metabolism. *J. Exp. Bot.* **57**, 2445–2453 (2006).

25. Zhang, Y. *et al.* An ETHYLENE RESPONSE FACTOR-MYB Transcription Complex Regulates Furaneol Biosynthesis by Activating QUINONE OXIDOREDUCTASE Expression in Strawberry. *Plant Physiol.* **178**, 189–201 (2018).

26. Sasaki, K., Takase, H., Kobayashi, H., Matsuo, H. & Takata, R. Molecular cloning and characterization of UDP-glucose: Furaneol glucosyltransferase gene from grapevine cultivar Muscat Bailey A (Vitis labrusca × V. vinifera). *J. Exp. Bot.* **66**, 6167–6174 (2015).

27. Zhong, S. *et al.* High-Throughput Illumina Strand-Specific RNA Sequencing Library Preparation. *Cold Spring Harb. Protoc.* **2011**, 940–948 (2011).

28. Wang, L. *et al.* A low-cost library construction protocol and data analysis pipeline for illumina-based strand-specific multiplex RNA-seq. *PLoS One* **6**, e26426 (2011).

29. Xu, H. *et al.* FastUniq: A Fast De Novo Duplicates Removal Tool for Paired Short Reads. *PLoS One* **7**, e52249 (2012).

30. Bolger, A. M., Lohse, M. & Usadel, B. Trimmomatic: A flexible trimmer for Illumina sequence data. *Bioinformatics* **30**, 2114–2120 (2014).

31. Marçais, G. & Kingsford, C. A fast, lock-free approach for efficient parallel counting of occurrences of k-mers. *Bioinformatics* **27**, 764–770 (2011).

32. Liu, B. *et al.* Estimation of genomic characteristics by analyzing k-mer frequency in de novo genome projects. *arXiv* **http://arx**, (2013).

33. Gnerre, S. *et al.* High-quality draft assemblies of mammalian genomes from massively parallel sequence data. *Proc. Natl. Acad. Sci.* **108**, 1513–1518 (2011).

34. Kajitani, R. *et al.* Efficient de novo assembly of highly heterozygous genomes from whole-genome shotgun short reads. *Genome Res.* **24**, 1384–1395 (2014).

35. Kurtz, S. *et al.* Versatile and open software for comparing large genomes. *Genome Biol.* **5**, R12 (2004).

36. Edgar, R. C. & Myers, E. W. PILER: Identification and classification of genomic repeats. *Bioinformatics* **21**, i152–i158 (2005).

37. Xu, Z. & Wang, H. LTR-FINDER: An efficient tool for the prediction of full-length LTR retrotransposons. *Nucleic Acids Res.* **w35**, W265–W268 (2007).

38. Price, A. L., Jones, N. C. & Pevzner, P. A. De novo identification of repeat families in large genomes. *Bioinformatics* i351–i358 (2005) doi:10.1093/bioinformatics/bti1018.

39. Holt, C. & Yandell, M. MAKER2: An annotation pipeline and genome-database management tool for second-generation genome projects. *BMC Bioinformatics* **12**, (2011).

40. Stanke, M., Tzvetkova, A. & Morgenstern, B. AUGUSTUS at EGASP: using EST, protein and genomic alignments for improved gene prediction in the human genome. *Genome Biol.* (2006) doi:10.1186/gb-2006-7-s1-s11.

41. Korf, I. Gene finding in novel genomes. *BMC Bioinformatics* (2004) doi:10.1186/1471-2105-5-59.

42. Grabherr, M. G. . *et al.* Trinity: reconstructing a full-length transcriptome without a genome from RNA-Seq data. *Nat. Biotechnol.* (2011) doi:10.1038/nbt.1883.Trinity.

43. Li, W. & Godzik, A. Cd-hit: A fast program for clustering and comparing large sets of protein or nucleotide sequences. *Bioinformatics* (2006) doi:10.1093/bioinformatics/btl158.

44. Jones, P. *et al.* InterProScan 5: Genome-scale protein function classification. *Bioinformatics* **30**, 1236–1240 (2014).

45. Bateman, A. *et al.* UniProt: The universal protein knowledgebase. *Nucleic Acids Res.* (2017) doi:10.1093/nar/gkw1099.

46. Kim, D. *et al.* TopHat2: accurate alignment of transcriptomes in the presence of insertions, deletions and gene fusions. *Genome Biol.* **14**, R36 (2013).

47. Trapnell, C. *et al.* Differential gene and transcript expression analysis of RNA-seq experiments with TopHat and Cufflinks. *Nat. Protoc.* **7**, 562–578 (2012).

48. Anders, S., Pyl, P. T. & Huber, W. HTSeq-A Python framework to work with high-throughput sequencing data. *Bioinformatics* (2015) doi:10.1093/bioinformatics/btu638.

**Supplementary Note 7**

**Supplementary figures**

**Supplementary Fig. 1** **Distribution of the 21-mer frequency in ‘Concord’ genome sequencing reads.**

**
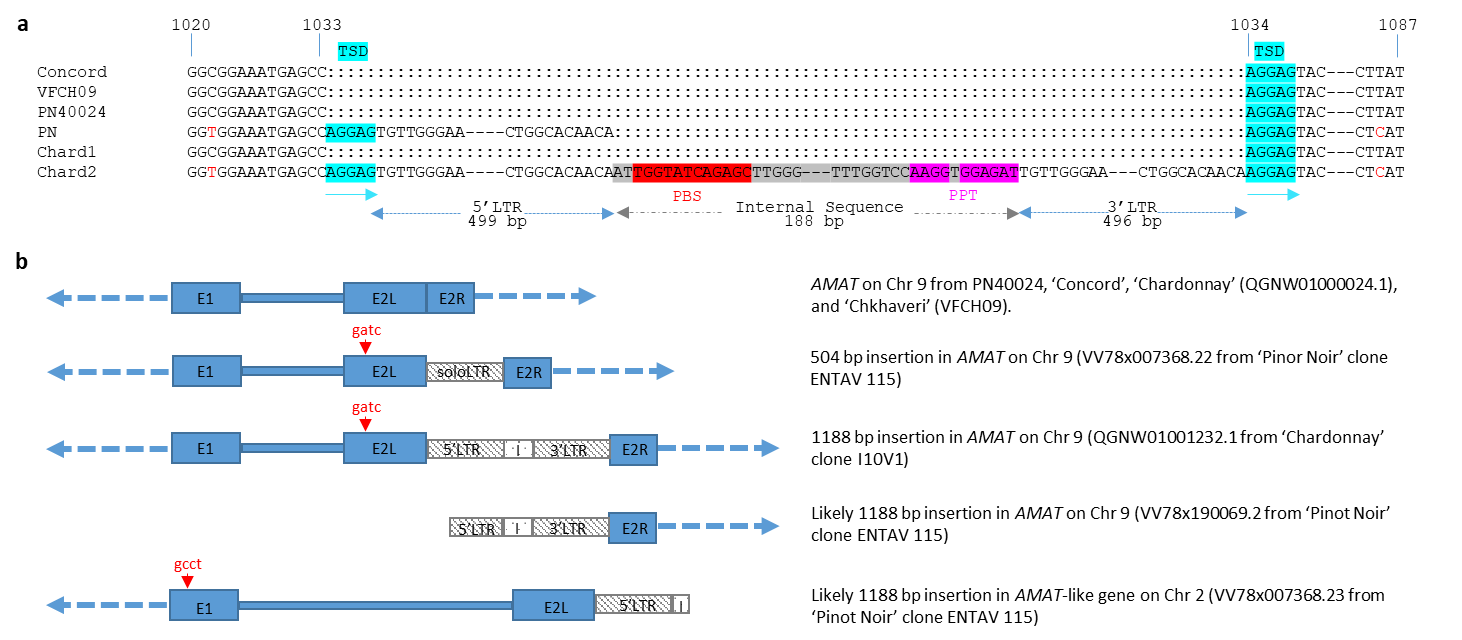
**

**Supplementary Fig. 2** ***TRIM insertion in the exon 2 of AMAT and AMAT-like genes.***

**(a)** Exon 2 of *AMAT* border sequences with the TRIM insertion. Sequences of ‘Concord’ and PN40024 are from this study and the reference genome, respectively. Other sequences are from published contigs: VFCH09 from ‘Chkhaveri’ (Tabidze et al., 2017), PN from the contig VV78x007368.22 of ‘Pinot noir’ clone ENTAV 115 (Velasco et al., 2007), and Chard1 (contig QGNW01000024.1) and Chard2 (contig QGNW01001232.1) from ‘Chardonnay’ clone I10V1 (Roach et al., 2018). Positions were based on *AMAT* ORF with ‘A’ in ATG as position 1. 5 bp target site duplication (TSD) was highlighted in blue and marked by solid arrows. 5’LTR and 3’LTR are very similar with a 3-bp gap and 1 mismatch between them. The primer binding site (PBS) is highlighted in red and the polypurine tract motif (PPT) is highlighted in pink. Note that the two *AMAT* copies with TRIM insertions carried some unique SNPs (in red letters). (**b)** Schematic diagram of the *AMAT* gene or *AMAT*-like gene from different cultivars/contigs. Two types of insertion were found in the exon 2 of *AMAT* on chromosome 9: one is 504 bp (soloLTR) (contig VV78x007368.22 from ‘Pino Noir’ ENTAV 115) and the other one is 1188 bp (complete TRIM) (contig QGNW01001232.1 from ‘Chardonnay’ clone I10V1 and likely contig VV78x190069.2 from ‘Pinot Noir’ clone ENTAV 115 only partially covering the *AMAT* gene). ‘Pinot Noir’ clone ENTAV 115 likely also has an *AMAT*-like gene on Chr 2 with a 1188 bp insertion (contig VV78x007368.23, the whole coding region was not covered in this contig) at the same position as in the *AMAT* on chromosome 9. The PN40024 reference genome does not have the exonic TRIM insertion in either *AMAT* or *AMAT*-like genes. It is likely that the TRIM was deleted in PN40024 or the *AMAT* copy is chimeric for the TRIM insertion in ‘Pinot Noir’ ENTAV 115 (some has complete TRIM, some had soloLTR). The *AMAT* on Chr 9 with TRIM insertion also had 4 bp (“gatc”) insertion in exon 2. The *AMAT*-like gene with the MITE insertion had 4 bp (“gcct”) in exon 1. E1, E2, and I stand for exon 1, exon 2, and internal sequence, respectively; E2L and E2R stand for left and right sides of exon 2, respectively.

**Supplementary Note 8**

**List of supplementary tables**

**Supplementary Table 1.** DNA libraries for 'Concord' genome shotgun assembly.

**Supplementary Table 2.** Statistics of assembly and annotation for the ‘Concord’ genome.

**Supplementary Table 3.** 'Concord' RNA-Seq libraries used for 'Concord' genome annotation.

**Supplementary Table 4.** Summary of collinear blocks between ‘Concord’ and the *V. vinifera* PN40024 reference genome.

**Supplementary Table 5.** Statistics of repetitive sequences in 'Concord' and the *V. vinifera* PN40024 reference genome.

**Supplementary Table 6.** Annotated 'Concord' genes, their corresponding genes in the reference genome PN40024 version 2 (V2) and version 3 (V3), and 3-kb promoter comparisons.

**Supplementary Table 7.** Statistics of predicted gene models in the 'Concord' genome.

**Supplementary Table 8.** Functional annotation of the predicted genes in 'Concord'.

**Supplementary Table 9.** Summary of pair-wise 3-kb promoter comparisons between 'Concord' and PN40024.

**Supplementary Table 10.** SNP concordance of 'Concord' with 'Catawba', 'Semillon', *V. amurensis* and four *V. labrusca* accessions.

**Supplementary Table 11.** RNA-Seq libraries of berry samples at three developing stages for 'Concord' and four *V. vinifera* cultivars.

**Supplementary Table 12.** Genes differentially expressed between fruit developmental stages within ‘Concord’ or *V. vinifera*, and corresponding stages between 'Concord' and *V. vinifera*.

**Supplementary Table 13.** RNA-Seq expression profiles for the candidate genes related to 'foxy' aroma and some other traits of interest.

**Supplementary Table 14.** *AMAT* ORF comparisons among 'Concord', 'Catawba', 'Caco', 'Alba', 'Barry', 'Merlot', 'Pinot noir', 'Chardonnary' obtained from this study and some *V. vinifera* cultivars published online.

**Supplementary Table 15.** *AMAT* promoter sequence comparisons among 'Concord', *V.labrusca* hybrids cloned from this study and *V. vinifera* cultivars published online.

**Supplementary Table 16.** *AMAT* expression, MA accumulation and status of various *AMAT* indels in 50 germplasm accessions.

**Supplementary Table 17.** Summary of *AMAT* indel screening in the USDA *Vitis* germplasm.

**Supplementary Table 18.** Online survey results of indels in the *AMAT* promoter and coding regions.

**Supplementary Table 19.** Distribution of the soloLTR in the*AMAT* gene in the PN40024 reference genome.

**Supplementary Table 20.** Materials for RNA-Seq and genomic libraries.

**Supplementary Table 21.** Primers for cloning, qRT-PCR and indel screening in this study.
